# Mechanism of Action of a Chinese Herbal Compound Containing Quercetin, Luteolin, and Kaempferol in the Treatment of Vitiligo Based on Network Pharmacology and Experimental Verification

**DOI:** 10.1155/2022/7197533

**Published:** 2022-12-16

**Authors:** Ziqian Xu, Yihui Xie, Jun Song, Juntao Huang, Weimin Shi

**Affiliations:** ^1^Department of Dermatology, Ningbo First Hospital, Zhejiang University, Ningbo, China; ^2^Department of Dermatology, Shanghai General Hospital, Shanghai Jiao Tong University School of Medicine, Shanghai, China; ^3^Department of Otolaryngology Head and Neck Surgery, Ningbo Medical Center (Ningbo Lihuili Hospital), The Affiliated Lihuili Hospital of Ningbo University, Ningbo, China

## Abstract

**Objective:**

This study aimed to explore the mechanisms of Baishi tablets (BSTs) in the treatment of vitiligo through network pharmacology-based identification and experimental validation.

**Methods:**

In brief, the compounds and related targets of BST were extracted from the TCMSP database, and disease information was obtained from the OMIM, GeneCards, PharmGkb, TTD, and DrugBank databases. A Venn diagram was generated to visualize the common targets of BST and vitiligo. GO and KEGG analyses were performed to explore the potential biological processes and signaling pathways. The PPI network and core gene subnetwork were constructed using STRING and Cytoscape software. In addition, the measurement of apoptosis in PIG1 cells and intracellular reactive oxygen species were measured using quercetin (QU), luteolin (LU), and kaempferol (KA) to protect melanocytes from oxidative stress.

**Results:**

A total of 55 compounds with 236 targets and 1205 vitiligo-related genes were obtained from the TCMSP database. GO and KEGG analyses were performed to explore the potential biological processes and signaling pathways, revealing that BST may cure vitiligo by influencing the biological processes of cellular oxidative stress and related signaling pathways. A critical subnetwork was obtained with 13 core genes by analyzing the PPI network, which includes *HMOX1*, *CXCL8*, *CCL2*, *IL6*, *MAPK8*, *CASP3*, *PTGS2*, *AKT1*, *IL1B*, *MYC*, *TP53*, *IFNG*, and *IL2*. Furthermore, a molecular docking analysis was conducted to simulate the combination of compounds and gene proteins, reflecting that QU, LU, and KA can strongly bind the core genes. Through a series of experimental validations, we found that QU, LU, and KA could attenuate H_2_O_2_-induced apoptosis in melanocytes. Further evidence revealed that QU, LU, and KA could enhance the scavenging of intracellular reactive oxygen species (ROS).

**Conclusion:**

Based on the results of network pharmacology analysis and experimental verification, QA, LU, and KA can be utilized to protect PIG1 cells by inhibiting oxidative stress and reducing the intracellular level of ROS. This may explain the underlying mechanism of BST therapy and provide a novel strategy for the treatment of vitiligo.

## 1. Introduction

Vitiligo is an acquired, chronic depigmenting disorder of the skin. It results from the selective destruction of melanocytes [1]. Approximately 0.5%–1% of the global population is affected by vitiligo. Vitiligo prevalence is not associated with sex or ethnicity [1–3]. Vitiligo is considered to be a multifactorial disease. In addition to genetic and environmental factors, other factors (autoimmune, neural, and oxidative stress) have been suggested to have an effect on vitiligo [4].

Management of vitiligo includes the topical use of glucocorticoids, calcineurin inhibitors, and phototherapy. A small number of patients who meet the indications can also choose surgical transplantation of pigments or decolorization treatment [5]. However, due to the complexity of the primary pathogenesis of vitiligo, the treatment methods stated above cannot cure it; they provide only short-term benefits, and the long-term effects are often unsatisfactory. Moreover, such treatments often introduce toxic side effects (e.g., skin atrophy, phototoxic reactions, and skin cancer).

Traditional Chinese medicine (TCM) is a powerful and far-reaching system of medical treatment. From the viewpoint of compatibility of TCM and the interaction of various traditional Chinese medicines, TCM formulations have relatively low toxicity and few side effects and have shown curative effects in the treatment of several diseases [6]. Studies have shown that TCM formulations have great potential in vitiligo treatment [7, 8].

Baishi tablets (BSTs) consist primarily of Chaihu (bupleurum), Chishao (red peony), Zhixiangfu (rhizome cyperi), Baishao (white peony), and Zhishi (citrus aurantium). BST can replenish qi, promote stagnation, aid blood circulation and detoxification, and dispel wind. The dermatology department of our institution has used BST to treat vitiligo for many years, and excellent outcomes have been achieved. Our research group conducted a clinical randomized trial in 2015, and the Vitiligo Disease Activity (VIDA) score of the patients treated with BST was significantly reduced [9]. Nevertheless, the components of TCM formulations have a wide range of functions and complex active ingredients, and the targets and mechanisms of their regulation have not been fully studied.

Network pharmacology is a promising method that combines pharmacology and computer science to construct and visualize the interaction network of multiple genes, targets, and signaling pathways. It is highly suitable for researching drugs with complex ingredients (e.g., TCM formulations) and is a cost-effective method of drug development [10–12].

**Figure 1:**
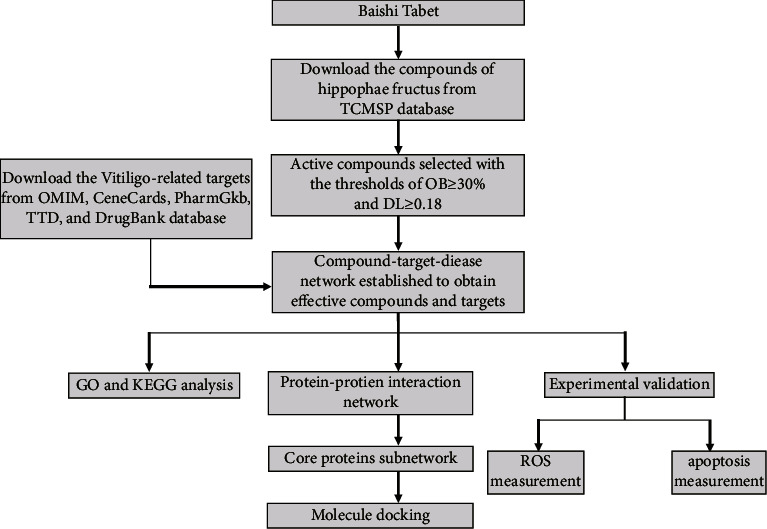
The flowchart for the mechanism exploration of the Baishi tablet. OB, oral bioavailability; DL, drug likeness. GO, gene ontology; KEGG, Kyoto encyclopedia of genes and genomes.

Herein, we applied network pharmacology to identify the active ingredients and examine the core targets and signaling pathways of BST for vitiligo treatment. We also undertook molecular docking studies to ascertain how BST binds to its predicted targets. Subsequently, we conducted a series of *in vitro* experiments on immortalized human melanocytes. The flowchart of our study is shown in Figure 1.

## 2. Materials and Methods

### 2.1. Identification of the Active Compounds in BST and Related Target Genes

The compounds Chaihu, Chishao, Xiangfu, Baishao, and Zhishi were searched and obtained from the Traditional Chinese Medicine System Pharmacology Database (TCMSP; http://tcmspw.com/tcmsp.php/) [13], as were their related gene targets and other biological information. Specifically, to evaluate the characteristics of absorption, distribution, metabolism, and excretion, we used oral bioavailability (OB) and drug likeness (DL) to filter candidate active compounds with thresholds of OB ≥ 30% and DL ≥ 0.18 [14]. Moreover, the targets of the active compounds were transformed into gene symbols *via* the UniProt database (http://www.uniprot.org/) by limiting the species to *Homo sapiens* for further analyses [15].

### 2.2. Identifying the Target Genes Related to Vitiligo

Vitiligo-related targets were extracted by screening the Online Mendelian Inheritance in Man (OMIM; https://omim.org/) [16], Genecards (http://www.genecards.org/) [17], PharmGkb (http://www.pharmgkb.org/) [18], Therapeutic Target Database (TTD; http://db.idrblab.net/ttd/) [19] and Drugbank (http://www.drugbank.ca/) [20] databases using the keyword “vitiligo.” After removing duplicates, a vitiligo-related gene set was established by combining the search results.

### 2.3. Establishment of a Compound–Target–Vitiligo Network and Functional Analyses

Having prepared two sets of target lists for the gene targets of compounds and vitiligo-related targets, screening for drug–disease crossover was carried out. A Venn diagram was generated with R (R Institute for Statistical Computing, Vienna, Austria) using the Venn Diagram package to show the intersection sets. A compound–target–disease network diagram was established using Cytoscape 3.8.0 (https://cytoscape.org/) to show the relationship among vitiligo, BST, and the related gene targets [21].

Subsequently, analyses of functional enrichment and enrichment of signaling pathways were undertaken using the gene ontology (GO; http://geneontology.org/) and Kyoto Encyclopedia of Genes and Genomes (KEGG; http://www.genome.jp/kegg/) databases, respectively. The “clusterprofile” and “bioconductor” packages within R were employed to assess the biological process (BP), cellular component (CC), molecular function (MF), and key signaling pathways. Significantly enriched terms were identified, and *p* < 0.05 and *q* < 0.05 indicated a strong association with related BPs [22, 23].

### 2.4. Protein–Protein Interaction (PPI) Networks and Critical Subnetworks

The Search Tool for the retrieval of interacting genes/proteins (STRING) (http://string-db.org/) database [24] was used to identify the biological interactions among the potential gene targets. Intersecting PPIs were obtained through the intersecting gene sets of BST and vitiligo. After importing the results of PPIs into Cytoscape, a diagram of critical subnetworks was established, and core genes were investigated using the CytoNca plugin [25]. Eligible genes were selected if each score was higher than the median value of betweenness, closeness, degree, the eigenvector, the local average connectivity-based method, and network scores. After displaying this analytical process twice, the final results of the core genes were utilized to establish a critical subnetwork.

### 2.5. Molecular Docking

Compounds with the top-three highest numbers of related critical genes and their common core genes were selected for molecular docking. After downloading the two-dimensional (2D) molecular structure of ligands from the PubChem database (https://pubchem.ncbi.nlm.nih.gov/) [26], the 3D structure with the minimum energy was calculated and exported *via* ChemBio 3D (http://www.adeptscience.co.uk/products/lab/chembio3d/). Moreover, the 3D structure of the receptor proteins encoded by the core genes was searched in the UniProt database and downloaded from the Research Collaboratory for Structural Bioinformatics Protein Database (https://www.rcsb.org/) [27].

After preparing the files for the 3D structure, the receptor proteins were dehydrated, and ligands were removed using PyMOL (https://pymol.org/2/). AutoDock (https://autodock.scripps.edu/) was utilized to modify the receptor protein as well as carry out the hydrogenation and charging calculations of proteins [28]. Subsequently, the parameters of the docking site of the receptor protein were set to include the sites of the active pocket. Molecular docking between compounds and receptors was investigated *via* Vina within AutoDock [29].

### 2.6. Cells and Cell Culture

An immortalized human melanocyte cell line (PIG1) was purchased from the American Type Cell Collection (Manassas, VT, USA). PIG1 cells were cultured in Dulbecco's modified Eagle's medium (Gibco, Grand Island, NY, USA) supplemented with 10% fetal bovine serum (Gibco). PIG1 cells were cultured in a 37°C incubator in an atmosphere of 5% CO_2_.

### 2.7. Apoptosis Measurement

PIG1 cells were cultured in 60 mm petri dishes after treatment with 50 *μ*mol/L QU, LU, or KA for 24 h. Then, H_2_O_2_ (final concentration: 1 mmol/L) was added to each well, and incubation was undertaken for an additional 2 h. Simultaneously, we set up simple compound-treatment groups and a control group (without any treatment). After the previous treatment, we digested PIG1 cells with EDTA-free trypsin, collected them in tubes, washed them twice with phosphate-buffered saline (PBS), and resuspended them in PBS. According to the protocol, PIG1 cells were stained with an Annexin V-FITC Apoptosis Detection Kit (Liankebio, Hangzhou, China) and detected by flow cytometry. FlowJo (http://www.flowjo.com/) was used to measure the percentage of apoptotic cells.

### 2.8. Measurement of Levels of Intracellular Reactive Oxygen Species (ROS)

QU, LU, and KA were purchased from Aladdin (purity ≥98.5%; Shanghai, China). QU, LU, and KA were dissolved in dimethyl sulfoxide (DMSO; Millipore Sigma‒Aldrich, Burlington, Massachusetts MA, USA) for further use. PIG1 cells were cultured in six-well plates after being treated with different concentrations (25 or 50 *μ*mol/L) of QU, LU, and KA for 24 h. H_2_O_2_ was added (final concentration: 1 mmol/L) to each well and incubated for an additional 2 h. After the corresponding treatment, we washed cells twice with serum-free medium. According to product instructions, cells were stained with dichlorodihydrofluorescein diacetate using a reactive oxygen species kit (Shanghai Biyuntian Biotechnology, Shanghai, China) and photographed under an inverted fluorescence microscope (Olympus, Tokyo, Japan).

### 2.9. Statistical Analyses

The data are the mean ± SD. Statistical analyses were carried out using Prism 7.0 (GraphPad, San Diego, CA, USA) or SPSS 22.0 (IBM, Armonk, NY, USA). The Student's t- test or one-way analysis of variance was used for multiple group comparisons. The experiments were repeated at least three times. *p* < 0.05 was considered significant.

## 3. Results

### 3.1. Active Compounds and Potential Targets

**Figure 2:**
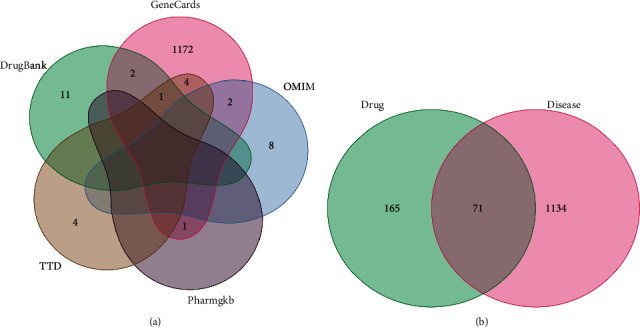
Identification of the drug-target interaction. (a) Venn diagram of vitiligo-related genes. (b) Venn diagram of gene intersections between BST and vitiligo BST, Baishi tablet.

Using the criteria of DL ≥ 0.18 and OB ≥ 30%, 55 main and efficacious compounds of the five herbs were retrieved and selected (Supplementary Table 1). Subsequently, the compound-related targets were annotated into a gene-symbol set using the UniProt database. After removing duplicates, a set of 1205 vitiligo-related targets (Supplementary Table 2A) were established by extraction from the OMIM, GeneCards, PharmGkb, TTD, and DrugBank databases (Figure 2(a), Supplementary Table 2B). Moreover, an intersection of the compound targets and vitiligo-related genes, which contained 71 target proteins (Supplementary Table 2C**)**, was obtained (Figure 2(b)).

### 3.2. Network Analysis of Targets

**Figure 3:**
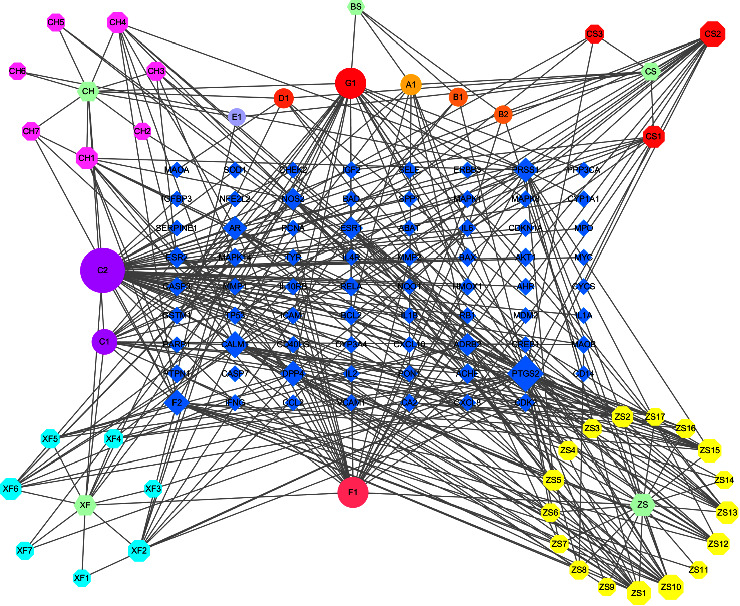
BST-compounds-genes-vitiligo network diagram. The annotation of compounds is summarized in Supplemental Table 1. BST, Baishi tablet.

A compound–disease–target interaction network (Figure 3) was visualized using the Cytoscape to reflect the relationship among the compounds in BST, vitiligo, and their intersecting genes. The number of possible efficacious compounds of BST related to vitiligo treatment was 43. The top five active ingredients influencing the most genes were QU (50 genes), LU (25 genes), KA (24 genes), isorhamnetin (15 genes), and baicalein (15 genes). The top five related gene proteins in the intersecting genes were *PTGS2* (38 compounds), *CALM1* (20 compounds), *DPP4* (18 compounds), *F2* (18 compounds), and *PRSS1* (16 compounds).

### 3.3. Enrichment Analyses

**Figure 4:**
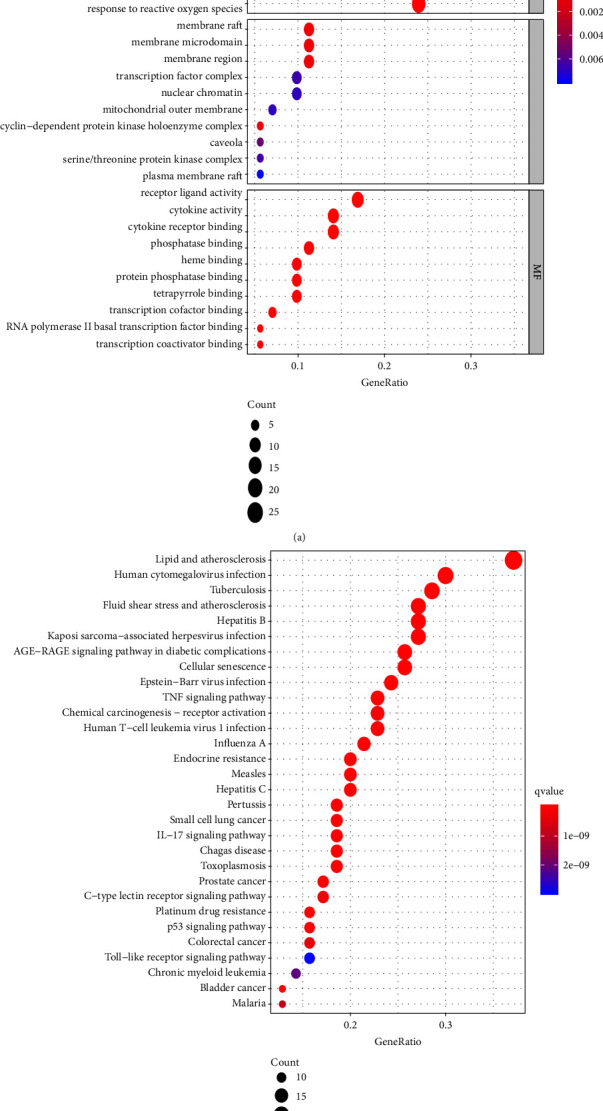
GO (a) and KEGG (b) enrichment analyses of the target genes. GO, gene ontology; KEGG, Kyoto Encyclopedia of Genes and Genomes.

Analyses of functional enrichment using the GO database revealed the underlying BPs, CCs, and MFs of the 71 target genes. Using *p* < 0.05 and *q* < 0.05 as criteria, 2112 significantly enriched GO terms were obtained: 1975 BPs, 26 CCs, and 111 MFs. The top 10 terms is shown in Figure 4(a).

The top 10 BPs were responses to metal ions; nutrient levels; lipopolysaccharide; molecules of “bacterial origin,” “radiation,” “oxidative stress,” “aging,” “antibiotics,” “drugs,” and “reactive to oxygen species.”

The top 10 CCs were “cyclin-dependent protein kinase holoenzyme complex,” “membrane raft,” “membrane microdomain,” “membrane region,” “caveola,” “serine/threonine-protein kinase complex,” “transcription factor complex,” “nuclear chromatin,” “mitochondrial outer membrane,” and “plasma membrane raft.”

The top 10 MFs were “cytokine activity,” “cytokine receptor binding,” “receptor ligand activity,” “phosphatase binding,” “transcription cofactor binding,” “RNA polymerase II basal transcription factor binding,” “heme binding,” “protein phosphatase binding,” “tetrapyrrole binding,” and “transcription coactivator binding.”

Using *p* < 0.05 and *q* < 0.05 as criteria, analyses of signaling-pathway enrichment using the KEGG database were performed. We found that 165 potential signaling pathways were enriched (Supplementary Table 3), and the top 30 signaling pathways are shown in Figure 4(b). Bubble plots demonstrated that these gene targets affected signaling pathways related to the “biological process of oxidative stress such as lipid and atherosclerosis,” “AGE-RAGE signaling pathway in diabetic complications,” “fluid shear stress,” and “atherosclerosis.”

### 3.4. PPI Diagram and Core Subnetwork

**Figure 5:**
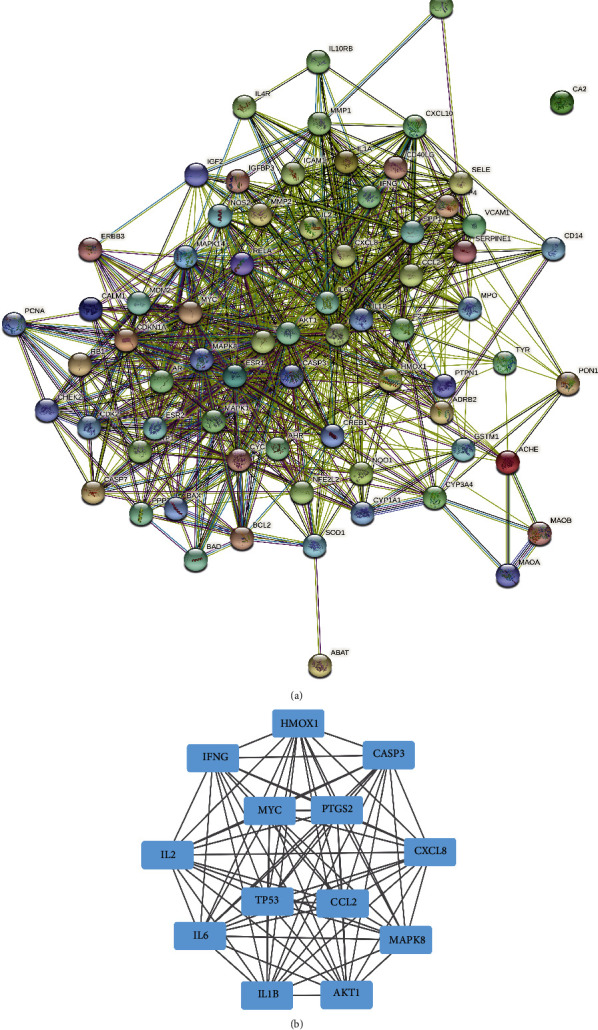
Protein-protein interaction network for BST in the treatment of vitiligo and core targets. (a) Protein-protein interactions among the 71 genes. Network nodes represent proteins, and edges represent protein-protein associations. (b) Core gene subnetwork of 71 overlapping genes.

Seventy-one overlapping genes associated with vitiligo and BST were inputted into the STRING database, and a PPI network diagram was established after selecting *Homo sapiens*. The PPI network contained 71 nodes and 821 edges (Figure 5(a), Supplementary Table 4). After importing the results from the PPI network into Cytoscape and using the CytoNCA plugin, these related genes were identified twice to establish a core-gene subnetwork. The median values of betweenness, closeness, degree, eigenvector, local average connectivity-based method, and network scores in the calculations were 17.06899034, 0.584745763, 22.5, 0.109364353, 15.794871795, and 18.691161565, respectively, in the first identification and 5.78362403, 0.8170634925, 22.5, 0.180951178, 18.09090909, and 20.229612495, respectively, in the second identification. A further core subnetwork containing 13 nodes and 77 edges was obtained. These 13 core gene targets were *HMOX1*, *CXCL8*, *CCL2*, *IL6*, *MAPK8*, *CASP3*, *PTGS2*, *AKT1*, *IL1B*, *MYC*, *TP53*, *IFNG*, *and IL2* (Figure 5(b)). The results from the analyses of functional enrichment and enrichment of signaling pathways using the GO and KEGG databases, respectively, revealed that the core genes were involved in the “cellular response to oxidative stress” and had critical roles in signaling pathways. Detailed information on these compounds is summarized in Supplementary Table 5.

### 3.5. Molecular Docking

**Figure 6:**
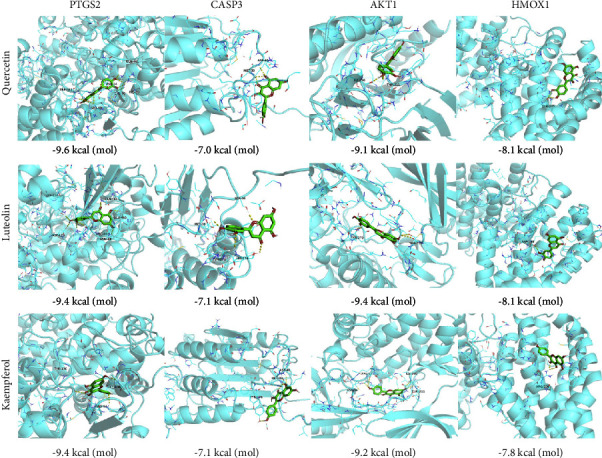
Virtual molecular docking results.

Referring to the results of the core gene network, we selected the top-three compounds (QU, LU, and KA) that influenced most vitiligo-related genes as ligands. Then, we conducted molecular docking on these core genes. According to our previous analyses, QU influenced 12 core genes (*PTGS2*, *AKT1*, *IL6*, *CASP3*, *TP53*, *HMOX1*, *MYC*, *IL1B*, *CCL2*, *CXCL8*, *IL2*, and *IFNG*), LU affected eight core genes (*PTGS2*, *AKT1*, *IL6*, *CASP3*, *TP53*, *HMOX1*, *IL2*, and *IFNG*) and KA regulated five core genes (*PTGS2*, *AKT1*, *CASP3*, *MAPK8*, and *HMOX1*). Subsequently, an additional calculation was made to simulate the molecular docking of three compounds with four common protein receptors: *PTGS2* (protein database (PDB) code: 5KIR), *AKT1* (PDB code: 6HHG), *CASP3* (PDB code: 3PD0) and *HMOX1* (PDB code: 1N45). The results of molecular docking and affinity values are listed in Figure 6. A greater absolute value for the docking affinity indicates a stronger binding ability between the active site of a protein receptor and a compound. The docking results indicated that QU, LU, and KA could enter and bind the active pocket of the four core target proteins, could form hydrogen bonds with amino acid residues, and exhibited high binding affinity.

### 3.6. Experimental Validation

#### 3.6.1. QU, LU, And KA Reduced H_2_O_2_-Induced PIG1 Apoptosis

**Figure 7:**
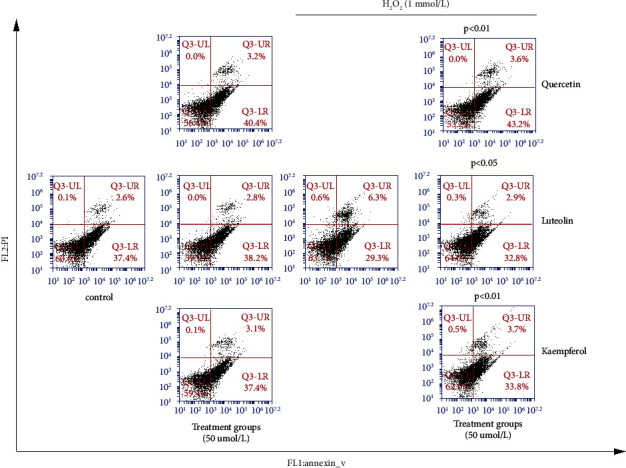
QU, LU, and KA attenuate H_2_O_2_-induced apoptosis in PIG1 cells. The cells were pretreated with QU, LU, and KA 50 *μ*mol/L for 24 h; then, the cells were treated with H_2_O_2_ (final concentrations: 1.0 mmol/L) for 2 (h) In addition, we set up QU, LU, KA-treated (50 *μ*mol/mL), pure-H_2_O_2_-treated (1.0 mmol/L), and control groups.

We wished to verify the prediction results of the previous compound–disease–target interaction network. We screened out the three compounds with the most extensive targets for vitiligo for experimental verification: QU, LU, and KA. The role of QU, LU, and KA in melanocytes under oxidative stress was explored by treating PIG1 cells with H_2_O_2_ to mimic the environment of cellular oxidative stress. We used flow cytometry to measure H_2_O_2_-induced apoptosis in PIG1 cells. H_2_O_2_ induced apoptosis, but pretreatment with QU, LU, or KA significantly reduced apoptosis. PIG1 cells treated with QU, LU, or KA alone did not induce significant apoptosis. There was a significant difference in the percent apoptosis between the pure H_2_O_2_-treated group and compound-pretreated groups (*p* < 0.05 and *p* < 0.01) (Figure 7).

#### 3.6.2. QU, LU, And KA Scavenged H_2_O_2_-Induced Intracellular ROS in PIG1 Cells

**Figure 8:**
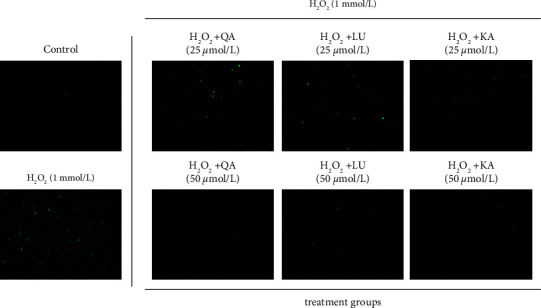
QU, LU, and KA scavenge H_2_O_2_-induced intracellular ROS in PIG1 cells. The cells were pretreated with QU, LU, and KA at different concentrations (25 and 50 *μ*mol/L) for 24 h and then treated with H_2_O_2_ (final concentrations: 1.0 mmol/L) for 2 h; we set up QU, LU, and KA-treated (25, 50 *μ*mol/mL), pure-H_2_O_2_-treated (1.0 mmol/L), and control groups.

First, PIG1 cells in the treatment groups were pretreated with different concentrations (25 or 50 *μ*mol/L) of QU, LU, or KA. Subsequently, PIG1 cells were treated with H_2_O_2_ to simulate the environment of cellular oxidative stress, and we measured ROS production by fluorescence staining. Compared with PIG1 cells in the pure-H_2_O_2_ environment, ROS levels in PIG1 cells decreased in all three groups that were pretreated with 25 or 50 *μ*mol/L of compounds. ROS levels decreased in the same treatment group with increasing concentrations of the compound, respectively (Figure 8). These results suggested that QU, LU, and KA could reduce ROS in melanocytes and protect melanocytes from oxidative stress to a certain extent.

## 4. Discussion

Vitiligo is caused by melanocyte destruction. Vitiligo pathogenesis could be due to heredity, autoimmunity, neurochemical factors, or oxidative stress [1, 2]. Oxidative stress may be the initial event leading to vitiligo development [30]. Melanocytes from patients with vitiligo are inherently defective and susceptible to oxidative stress [31]. ROS generation occurs during melanin synthesis by melanocytes. The stress status of melanocytes can also lead to an excessive accumulation of ROS, which results in changes to the antioxidant system. The imbalance of oxidative and antioxidant systems in vitiligo patients increases the sensitivity of melanocytes to external oxidants, thereby resulting in premature aging and apoptosis [32, 33]. Excessive accumulation of ROS can also cause cellular DNA damage and lipid peroxidation, which affect cellular function [34, 35]. Therefore, reducing oxidative stress in melanocytes should be a rational strategy for vitiligo treatment.

BST has a satisfactory effect in the clinical treatment of vitiligo. However, due to the complex components of TCM formulations, a more accurate and systematic study of their possible targets and mechanisms is needed. We used network pharmacology to explore the mechanism of action of BST for vitiligo treatment. QU, LU, KA, baicalein, nobiletin, and isorhamnetin were screened out as the main active ingredients of BST. QU, LU, and KA had the most extensive targets in vitiligo, so we selected them for experimental verification.

QU is a polyphenolic flavonoid found widely in onions, cabbage, apples, and tea [36, 37]. In recent years, scholars have revealed that QU has powerful antioxidant effects. It has a preventive effect on osteoporosis, certain tumor types, and certain cardiovascular diseases. The antioxidant activity of QU occurs mainly through the direct induction of glutathione (GSH) synthesis in the body. GSH acts as a hydrogen donor for redox reactions in the body, while superoxide dismutase captures O_2_ molecules and transforms them into H_2_O_2_, thereby having an antioxidant effect [38]. In addition, the -OH group in QU can bind to the active sites of oxidative enzymes, such as acetylcholinesterase and butyrylcholinesterase, to inhibit their activity and elicited an antioxidant effect [39]. QU can also regulate the NRFB, 5′ adenosine monophosphate-activated protein kinase, and mitogen-activated protein kinase signaling pathways [40, 41]. Studies have shown that QU increases the tyrosinase activity and synthesis of melanoma cells and normal melanocytes to promote melanogenesis [42]. QU has a weakening and protective effect on H_2_O_2_-induced endoplasmic reticulum stress in melanocytes [43]. Here, we demonstrated that a certain concentration of QU reduced the ROS level in human immortalized melanocytes induced by H_2_O_2_ and had a certain degree of protection against oxidative stress in melanocytes. Combined with the results of network pharmacology, we showed that QU had the most extensive binding targets in BST.

LU is a flavonoid found in vegetables and fruits and is used in Chinese herbal medicines [44, 45]. TCM formulations containing LU have been employed to treat high blood pressure, inflammation, and cancer [44]. LU can inhibit the release of interleukin (IL)8, a critical proinflammatory chemokine in vitiligo and may have the potential to treat vitiligo [46].

KA is a natural flavonoid found in tea, fruits, and vegetables. Lee and colleagues found that KA could inhibit ultraviolet B-induced expression of cyclooxygenase-2 (COX-2) release in mouse skin epidermal (JB6P^+^) cells and attenuate ultraviolet B-induced COX-2 release and activator protein-1 transcriptional activity [47]. KA can also improve the skin fibrosis induced by bleomycin by reducing oxidative stress and inflammation [48]. Our experimental study revealed that a certain concentration range of KA could reduce ROS levels and protect melanocytes.

Hence, QU, LU, and KA are flavonoids that have anti-inflammatory and antioxidant effects. Therefore, we speculated that they could affect the oxidative stress of melanocytes. We conducted experiments to verify that under oxidative stress (mimicked by H_2_O_2_ use), melanocyte apoptosis and ROS production were reduced under pretreatment by QU, LU, or KA. Therefore, BST may have a specific protective role in the oxidative stress pathway of melanocytes.

In the PPI network analysis of BST, *HMOX1*, *CXCL8*, *CCL2*, *IL6*, *MAPK8*, *CASP3*, *PTGS2*, *AKT1*, *IL1B*, *MYC*, *TP53*, *IFNG*, and *IL2* were screened out, all of which are core targets in the treatment of vitiligo. *PTGS2* is also known as *COX2*, which plays an essential part in producing prostaglandin (PG)E2 and is made by epidermal keratinocytes in response to ultraviolet radiation [49, 50]. PGE2 is essential for the proliferation and melanogenesis of melanocytes, the loss of which can lead to vitiligo. In addition, studies have shown that the functional polymorphisms of *COX2* affect the risk of vitiligo [51]. Heme oxygenase-1 (HMOX1) is the most highly induced antioxidant gene in H_2_O_2_-treated PIG1 cells. HMOX1 has been demonstrated to protect human melanocytes from oxidative damage via the E2-related factor 2 (Nrf2)-antioxidant response element (ARE) pathway [52]. AKT1 is a RAC-alpha serine/threonine protein kinase. The phosphorylation of AKT1 could promote the accumulation of *β*-catenin, thereby activating the microphthalmia-associated transcription factor and tyrosinase family, eventually leading to melanogenesis of melanocytes [53]. Overexpression of cellular tumor antigen P53 (TP53) could protect the pigmentation around hair follicles in vitiligo patients after ultraviolet-B treatment, change the migration ability of melanocytes, and improve pigmentation in vitiligo patients [54]. IL-6 is a vital immune factor involved in autoimmune inflammation in vitiligo. Its increased expression in serum and a skin lesion could trigger an immune response that targets and kills melanocytes and leads to vitiligo [1, 55]. These results suggest that QA, LU, and KA could protect melanocytes, promote melanogenesis, inhibit melanocyte death, and protect melanocytes from oxidative damage through various mechanisms.

Our study had three main limitations. First, experimentally validated targets were the predicted results of network pharmacology, but there were certain deviations compared with the actual targets. Second, we revealed that the main compounds QU, LU, and KA could protect melanocytes from oxidative stress; however, how they regulate targets and affect downstream signaling pathways to have a role in vitiligo treatment was not tested. Third, our study was based on network pharmacology, so the compounds with the most targets were selected for experimental verification according to the results of network pharmacology, but their concentration in BST could not be determined.

## 5. Conclusions

Under the prediction obtained using network pharmacology, we clarified the active compounds in BST and their main targets in vitiligo treatment. Based on network pharmacology and *in vivo* experiments, QA, LU, and KA can be utilized to protect PIG1 cells. This phenomenon was achieved thanks to the inhibition of oxidative stress by reducing the intracellular level of ROS. This may explain the underlying mechanism of action of BST therapy and could provide a novel strategy for the treatment of vitiligo.

## Data Availability

The datasets presented in this study are openly available from TCMSP, GeneCardS, OMIM, TTD, PharmGkb, and DrugBank belong to public databases. The data used to support the findings of this study are available from the corresponding author upon request.

## Supplementary Materials

Supplementary Materials Supplementary Table 1. Compounds and related targets genes of Baishi Tablet. Supplementary Table 2A. Summarized vitiligo-related genes. Supplementary Table 2B. Vitiligo-related genes from five database respectively. Supplementary Table 2C. The interacting genes between vitiligo and Baishi tablet. Supplementary 3. Go enrichment analysis results. Supplementary 4. Protein-protein interaction network results. Supplementary 5. Description about compound-vitiligo-target network.
